# The CRISPR/Cas System: A Customizable Toolbox for Molecular Detection

**DOI:** 10.3390/genes14040850

**Published:** 2023-03-31

**Authors:** Yuxuan He, Wei Yan, Likun Long, Liming Dong, Yue Ma, Congcong Li, Yanbo Xie, Na Liu, Zhenjuan Xing, Wei Xia, Feiwu Li

**Affiliations:** Institute of Agricultural Quality Standard and Testing Technology, Jilin Academy of Agricultural Sciences, Changchun 130033, Chinalonglikun@126.com (L.L.);

**Keywords:** CRISPR/Cas system, point-of-care testing, molecular detection, application

## Abstract

Clustered regularly interspaced short palindromic repeats (CRISPR) and their associated proteins (Cas) are promising molecular diagnostic tools for rapidly and precisely elucidating the structure and function of genomes due to their high specificity, programmability, and multi-system compatibility in nucleic acid recognition. Multiple parameters limit the ability of a CRISPR/Cas system to detect DNA or RNA. Consequently, it must be used in conjunction with other nucleic acid amplification techniques or signal detection techniques, and the reaction components and reaction conditions should be modified and optimized to maximize the detection performance of the CRISPR/Cas system against various targets. As the field continues to develop, CRISPR/Cas systems have the potential to become an ultra-sensitive, convenient, and accurate biosensing platform for the detection of specific target sequences. The design of a molecular detection platform employing the CRISPR/Cas system is asserted on three primary strategies: (1) Performance optimization of the CRISPR/Cas system; (2) enhancement of the detection signal and its interpretation; and (3) compatibility with multiple reaction systems. This article focuses on the molecular characteristics and application value of the CRISPR/Cas system and reviews recent research progress and development direction from the perspectives of principle, performance, and method development challenges to provide a theoretical foundation for the development and application of the CRISPR/CAS system in molecular detection technology.

## 1. Introduction

**Figure 1:**
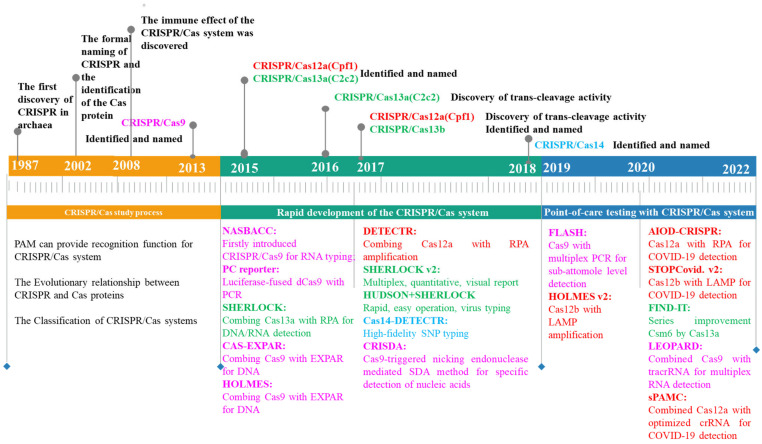
The CRISPR/Cas system development timeline. The timeline is constructed based on the publication date, which covers the discovery, identification, and application of Cas protein. Pink: CRISPR/Cas9 system; red: CRISPR/Cas12 system; green: CRISPR/Cas13 system; blue: CRISPR/Cas14 system. dCas9: Nuclease-deactivated Cas9; NASBACC: nucleic acid sequence-based amplification-CRISPR cleavage; PC reporter: paired dCas9; SHERLOCK: Specific High Sensitivity Enzymatic Reporter Unlocking; HUDSON: Heating Unextracted Diagnostic Samples to Obliterate Nucleases; CAS-EXPAR: CRISPR/Cas9 triggered isothermal exponential amplification reaction; HOLMES: one-HOur Low-cost Multipurpose highly Efficient System; DETECTR: DNA Endonuclease-targeted CRISPR Trans Reporter; FLASH: Finding Low Abundance Sequences by Hybridization; AIOD-CRISPR: All-In-One Dual CRISPR–Cas12a; STOP COVID: SHERLOCK Testing in One Pot COVID-19; FIND-IT: Fast Integrated Nuclease Detection In Tandem; LEOPARD: Leveraging Engineered tracrRNAs and On-target DNAs for Parallel RNA Detection; sPAMC: suboptimal Protospacer Adjacent Motifs of Cas12a-based test; SDA: Strand Displacement Amplification; RPA: Recombinase Polymerase Amplification; LAMP: Loop-mediated isothermal amplification; SNP: Single nucleotide polymorphism.

Since 2012, scientists have routinely used CRISPR to delete, mutate, replace, or add DNA sequences for the precise design and creation of new beneficial traits. Due to its low cost, simplicity of construction, ease of operation, and versatility, the CRISPR system has been dubbed “genetic magic scissors” by researchers, and it has been awarded the 2020 Nobel Prize in Chemistry. This revolutionary discovery created limitless opportunities for innovation and development throughout the entire field of biotechnology (Figure 1). Since 2017, CRISPR technology has grown rapidly in the field of molecular detection, with several advantages, including a rapid mode of operation, high accuracy, low cost, and low device dependence [1].

After ZFNs (zinc finger nucleases) and TALENs (transcription activator-like effector nucleases), the research community quickly developed the CRISPR system because of its simplicity, cost-effectiveness, and labor intensity. It was initially identified as short tandem repeats and spacer sequences near the alkaline phosphatase gene encoded in the *Escherichia coli* genome, but its synergistic function with the nearby Cas protein was not investigated until 2002 [2]. In its natural form, each CRISPR genomic locus is organized as an array of repeating sequences interspersed with variable sequences corresponding to invasive mobile genetic elements. These variable sequences are termed “spacers”. The defense role of a CRISPR-based system is achieved by spacers, which can be transcribed and processed into mature crRNA [3]. The CRISPR assays are flanked by multiple conserved protein-coding genes that function at different stages of CRISPR-mediated immunity and are usually prefixed with “CRISPR-associated” (Cas). Its immune function has three phases: Adaptation, expression, and interference [4]. At the adaptation stage, bacteria capture invading foreign nucleic acids via Cas proteins (Cas1 and Cas2) and integrate them into their CRISPR sequence to form a memory function, allowing them to initiate an immune response in the event of a subsequent invasion. In the expression phase, CRISPR sequences are subsequently transcribed into precursor crRNA (pre-crRNA) and trans-activating crRNA (tracrRNA). The pre-crRNA is then transformed into crRNA that is complementary to the sequences of foreign genes. In order to simplify genome editing applications, crRNA and tracrRNA can be artificially fused into a single-guide RNA (sgRNA) [5], which is used to recruit Cas protein. The sgRNA then directs the Cas protein to its foreign homologous nucleic acid target, activates the endonuclease activity of the Cas protein, and then performs an immune function by cleaving the target nucleic acid during the interference phase.

This review discusses the potential and limitations of exploiting the sensitivity and specificity of detection techniques from laboratory to environmental tests, based on the fundamental characteristics and functions of each CRISPR/Cas system, with an emphasis on the promising research in fields such as disease-related diagnostic methods, GMO detection, biosensor development, and other target detection. Finally, the fundamentals and most recent developments of rapid and accurate detection technology based on the CRISPR/Cas system are reviewed in order to provide new options and ideas for the development of molecular detection strategies.

## 2. Classification and Characterization of CRISPR Systems

Haft et al. [6] established the first simple classification of CRISPR/Cas systems in 2005 based on the evolutionary tree of the Cas1 protein and the differences in the sequence information of the Cas gene operon. In 2011, Makarova et al. [7] categorized the vast majority of CRISPR/Cas systems into types I (containing Cas3 protein), II (containing Cas9 protein), and III (containing Cas10 protein), with Cas1 and Cas2 genes serving as the core of the system. The few unclassifiable items are termed U-shaped. In 2015, Makarova et al. [8] analyzed 93 known CRISPR/Cas system protein families by developing a library of 394 position-specific scoring matrices (PSSM), in addition to two presumed new types, type IV (belonging to Class 1) and type V (belonging to Class 2) CRISPR/Cas systems. The type V system has a smaller protein size to type II [9,10] and type VI target RNA for cleavage [11]. The National Center for Biotechnology Information (NCBI) has recorded 13,116 complete archaea, and bacterial genomes as of 1 March 2019, and 7915 CRISPR/Cas loci were identified using BLAST. Based on the characterization of effect complexes and accessory Cas genes, as well as the structure of CRISPR/Cas loci, CRISPR/Cas systems are divided into 2 classes, 6 types and 33 subtypes [12] (Table 1). CRISPR/Cas types differ in their effects on the complex composition, target nucleic acid type, and cleavage outcomes due to differences in their molecular modes of action, resulting in a wide range of genetic applications. Currently, the majority of CRISPR/Cas systems used in molecular detection technology are Class 1 CRISPR/Cas3, Class 2 type II CRISPR/Cas9, type V CRISPR/Cas12, type VI CRISPR/Cas13, etc.

### 2.1. Class 1 and Its Derivatives

The proteins comprising the first class of CRISPR/Cas systems function as effector modules comprised of multiple Cas proteins, namely, Cas3 (sometimes fused with Cas2), Cas5–Cas8, Cas10, and Cas11, some of which form cooperative crRNA-binding complexes [13]. Consequently, they can be classified further as types I, III, and IV.

Presently, type I makes up the majority of the CRISPR/Cas system and contains a variety of defective subtypes. Cas1 and Cas2 are the most conserved proteins in the CRISPR/Cas system, and Cas1 is typically associated with other Cas genes and all type I, type II, the majority of type III-A systems, and some type III-B systems. During the adaptation phase of the CRISPR/Cas system, the Cas1–Cas2 complex incorporates new invader spacers into the CRISPR site as a molecular memory for pre-infection [14,15].

Cas4 participates in the selection and processing of spacer precursors, and this function depends on interactions with Cas1 or Cas2 [16]. Cas4–Cas1 recognizes and cleaves 3 ‘dangling protospacer adjacent motif (PAM) sequences in a sequence-specific manner, whereas the Cas1–Cas2 complex influence the cleavage of non-PAM sites by host factor nucleases. Furthermore, both subcomplexes are capable of interfering with spacers mediated by the processor by facilitating half-site integration [17]. Cas3, a second marker protein of the type I CRISPR system, possesses the HD phosphohydrolase and Superfamily2 (SF2) helicase domains. The Cas3 nuclease domain is recruited by a conformational change in the Cascade complex of the CRISPR system to form a single-chain effector that binds to the complementary chain, resulting in preferential cleavage of the complementary chain [18]. The characteristic helicase motifs (I, II, and VI) responsible for ATP binding and hydrolysis in the primary sequence of Cas3 predict that the protein has ATPase activity, which was significantly enhanced by the presence of single-stranded DNA (ssDNA) but not by double-stranded DNA (dsDNA) or RNA [19]. Notably, temperature regulates Cas3 nuclease activity, and temperature changes between 30 °C and 37 °C correspond to a conformational rearrangement of Cas3 associated with a single tryptophan residue (Trp-406) in Cas3 protein. Cas3 system Trp-406 is also essential for controlling the access of ssDNA to nuclease active sites [20].

### 2.2. Class 2 and Its Derivatives

The functional proteins of the Class2 system consist of single, multi-domain, crRNA-binding proteins that contain all of the essential components for nucleic acid cleavage. Multiple Class1 groups can collaborate to accomplish a task that only requires a single group to complete. This class of systems, which includes types II, V, and VI, is the preferred option for the construction of molecular detection platforms due to their higher gene editing efficiency and simpler reconfiguration [21].

The CRISPR/Cas9 system is typical of Type II systems. Cas9 protein, tracrRNA, and crRNA that can be inserted artificially into sgRNA constitute the system. The 5’ region of tracrRNA complements the repeat sequence of crRNA, and its function is to promote the maturation of crRNA. crRNA is capable of pairing with complementary bases on the target DNA to direct the binding of Cas9 to DNA. Cas9 nucleases contain two domains: RuvC-like (cross-over junction endodeoxyribonuclease) and HNH (His-Asn-His) when the CRISPR/Cas9 system moves to a specific target site and forms a cleavage complex. The RuvC-like and HNH domains have cleavage activity and are responsible for cleaving the target sequence and the complementary sequence of the target gene, respectively, according to [22]. The cleavage sites are 3–8 nt upstream of PAM sequence [23]. Double strand breaks (DSBs) are caused by cleavage and induce either homology-directed repair (HDR) or non-homologous end joining (NHEJ) in cells. These two mechanisms for gene repair allow for the insertion, deletion, or replacement of damaged DNA. Other CRISPR systems of the same type II as Cas9 are CRISPR/SpCas9, CRISPR/FnCas9, and CRISPR/SaCas9, all of which are found in the adaptive immune systems of *Staphylococcus aureus*.

The CRISPR/Cas12a (CRISPR/Cpf1) system in subtype V A is composed of two components: crRNA and Cas12a nuclease, which can recognize the PAM sequence (5′-TTN-PAMs) consisting of two or three consecutive thymine (T) bases at the 5′ end. By severing pre-crRNA(pre-crispr RNA), Cas12a nuclease is able to generate mature crRNA. Without the participation of tracrRNA, the target DNA was cut under the direction of crRNA. The Nuc domain on Cas12a nuclease plays an indirect role in the splicing process by aiding the RuvC domain, which splices the targeted DNA directly. The evolution of Cas9 and Cas12a initiated by the integration of a RuvC domain-encoding TnpB transposon with a stand-alone CRISPR array [24], as the functional platforms for subsequent insertions of additional domains, the conformational transitions of the RuvC domain and HNH domain will render Cas9 and Cas12a inactive for cleavage [25,26,27]. Similar to CRISPR/Cas12a, CRISPR/Cas12b is a functional protein of the CRISPR/Cas12 system. Comparable to the Cas12a system in terms of composition and mechanism of action, the AacCas12b system requires in vitro cleavage temperatures above 40 °C to maintain activity. Therefore, it cannot be utilized for mammalian cells. To identify the mesophilic Cas12b nucleases, 27 members of the Cas12b proteins within V-B loci were aligned using a BLAST search. AkCas12b and BhCas12b displayed strong DNA cleavage activity at 37 °C. The BhCas12b v4 mutant exhibited high specificity, excellent safety, and a broad genomic targeting range. Accordingly, we anticipate a new generation of safe and effective gene editing tools [28].

Cas14 protein (also known as Cas12f) is the shortest known Cas protein, containing between 400 and 700 amino acids. The three subtypes, viz., Cas14a, Cas14b, and Cas14c, are capable of both cis-cleavage (recognizes and cleaves nucleic acid sequences) and trans-cleavage (indiscriminately and non-specifically cleaves single-stranded DNA or RNA). When directed by sgRNA, they are able to cut ssDNA without a PAM site (tracrRNA:crRNA). Multiple CRISPR/Cas14 systems evolved independently, and while the Cas14 proteins exhibited substantial sequence diversity, all Cas14 proteins were bound together by the RuvC nuclease domain, which is typical of V-type CRISPR/Cas systems [29,30].

Although research on the V-type CRISPR/Cas system is still in its infancy, it has been implemented in numerous fields of molecular detection technology due to its low cost of detection, rapid reaction speed, and lack of restriction on the target sequence. For instance, the optimized CRISPR/Cas12 detection system can type multiple apple RNA viruses in under an hour [31]. CRISPR/Cas12a, in conjunction with sequence-specific crRNA and fluorescent probes, can be used to detect meat adulteration [32,33]. Cas12a-based virus typing technology can accurately screen influenza virus A, influenza virus B, and the COVID-19 virus during the COVID-19 outbreak [34,35]. CRISPR/Cas14a in conjunction with strand displacement amplification (SDA) can detect miR21 at concentrations as low as 680 fM in an hour, which is 2.86 times more sensitive than Cas12a [36].

Cas13 in type VI targets single-stranded RNA and is the first Cas protein discovered to recognize RNA specifically [37], with the ability to cleave ssRNA in both cis and trans orientations. Cas13a (C2c2), Cas13b, Cas13c, and Cas13d make up the majority of the Cas13 family [38,39,40,41]. Further investigation revealed that the system does not require tracrRNA, but rather a protospacer flanking site (PFS) similar to PAM that has an “adjunct cleavage effect” on RNA [11]. Due to the diminutive size of CRISPR/Cas13 proteins, they can be packaged into adeno-associated virus (AAV) vectors. In addition, they have a high delivery efficiency in mammalian systems. Several laboratories have been using Cas13 proteins to detect the COVID-19 virus [42], and are developing small-molecule drugs to combat the COVID-19 virus and other RNA viruses [43].

## 3. Mechanism of Molecular Detection Technology Based on CRISPR/Cas Systems

The technology for CRISPR-based molecular detection based on the following three system characteristics: (1) The specific binding to DNA or RNA; (2) the specific cleavage of target DNA or RNA; and (3) the trans-cleavage activity against ssRNA or ssDNA that is activated by specific sequence recognition. Certain subtypes of Cas12 and Cas13 exhibit indirect non-specific cleavage activity when guided by sgRNA. Once the target sequence has been identified, any encountered ssDNA or ssRNA can initiate the targeted cleavable activity, resulting in the degradation of the target molecule. This characteristic is also referred to as “collateral cleavage” and “trans-cleavage activity”. The target type is not constant in the detection procedure. Through transcription or reverse transcription, target DNA and RNA are capable of “mutual transfer” of the target type. The CRISPR/Cas system detects the presence of target nucleic acid based on the fluorescence groups of report molecules released by trans-cleavage activity [44].

### 3.1. Guidance RNA Biogenesis

The specificity and efficiency of Cas protein-mediated genome editing are determined by sgRNA properties such as the GC content, melting temperature, minimum free energy, and position-specific and non-specific nucleotide composition [45] (Table 2). The optimal sgRNA should have maximum target-guided efficiency while performing few or no cleavages in other regions of the genome. The following principles should guide the design of sgRNA: (1) The similarity between sgRNA and other sequences must be minimized, and there must be more than three mismatches between sgRNA and non-target sequences; (2) At least two of the mismatches must be in the seed region of the non-target sequence: (3) The mismatches must be continuous or separated by no more than 4 nt, and the GC content of the sgRNA must be below 35%. For a single base mismatch and a double base mismatch at different interspaces, the mismatch rate of the 5′ end of sgRNA was greater than that of the 3′ end, and different target sequences were sensitive to the exact location of the sgRNA mismatch. A significant decrease in Cas9 nuclease activity was observed when 3 or more mismatches were introduced. Even though the length of the spacer-derived segment of the sgRNAs is similar in both Cas9 (~20 nt) and Cas12a (~24 nt), the Cas12a endonucleases only require CRISPR RNA(crRNA) as guidance, and it uses a T-rice PAM with distinct features from Cas9 [46]; thus, the shortness of Cas12a crRNAs reduces the cost of chemical synthesis and improves the delivery efficiency compared to that of Cas9 due to the shorter repeat- and tracrRNA-derived segments [45].

### 3.2. Recognition of the PAM

Protospacer adjacent motif (PAM) recognition is indispensable for distinguishing autogenous DNA from non-autogenous DNA in prokaryotic hosts in type II and type V CRISPR/Cas systems. SpCas9, for instance, first recognizes the 5′-NGG-3′ site downstream of the target sequence and then forms an “R-loop” structure after crRNA binds to the target chain, which ultimately activates the cleaving activity of Cas9. Sequence-specific binding provides the foundation for the CRISPR/Cas9 system to capture [47] and label target genes in situ [48]. However, Cas12 recognizes AT-rich PAM regions, including 5′-TTTV-3′ (LbCas12, AsCas12a) and 5′-ATTN-3′ (BhCas12b). Cas12 is superior to Cas9 for cleaving DNA sequences containing a greater proportion of AT bases.

In the field of pathogen detection, candidate target sequences with suitable PAM sequences are easy to identify. However, it may be difficult to satisfy the Cas effector requirements for specific PAMs when the CRISPR system is used for SNP identification and other short-sequence detection. Located (+4) to (+7) downstream of PAM, Wang et al. discovered a 4-bp “core” sequence. The majority of mutations in the “core” sequence would reduce the efficiency of Cas9 and Cas12a for gene editing; therefore, this region was also an important determinant of targeting specificity [49].

In order to address the limitations of PAM, researchers have developed a series of gene editors with greater target compatibility and editing efficiency. For instance, spCas9 extends NGG to NG PAM sequences [50]. xCas9, developed by Hu et al., extended the PAM region from NGG to NGN, GAA, and GAT [51]. SpRY also expanded the PAM region to include NRN and NYN [52]. In addition, enAsCas12a was found to be suitable for multi-locus editing, with an editing efficiency approximately seven times higher than that of the majority of Cas12a homologs, as well as good targeting activity [53]. In addition, PAM sequences can be introduced into PCR or Loop-mediated isothermal amplification (LAMP) products by employing primers with PAM sites. This enables PAM-independent detection of Cas12 protein at nucleic acid sites [54,55]. PFS, unlike PAM, is a single base downstream of the protospacer that inhibits pairing between single-stranded RNA and the Spacer upstream base on crRNA. Thus, the effect of target cleavage mediated by some Cas13 proteins was modified, and the first base of PFS was changed to a non-G base, removing the restriction of PFS on Cas13 protein and allowing RNA editing, modification, and visualization [11].

### 3.3. Target DNA Binding and Cleavage

Although both Cas9 and Cas12 systems can target DNA, there are differences between them. Cas9, unlike Cas12, Cas14, and Cas13, is incapable of non-specifically cleaving nucleic acid probes following activation [48]. Secondly, Cas9 nuclease is applicable to a broad temperature range, whereas Cas12b nuclease has a higher temperature requirement; therefore, the optimal dissociation temperature for Cas12b nuclease is greater than 40 °C. When the reaction temperature was 37 °C, wild-type BhCas12b preferentially cut complementary DNA without forming double-strand breaks, resulting in a decrease in editing efficiency, which was hypothesized to be due to the difficulty of target DNA accessing the RuvC site of BhCas12b [56], as well as the difficulty of target DNA accessing the RuvC site of BhCas12b. By mutating BhCas12b, researchers increased the DNA’s affinity for the Cas mutant protein, thereby removing the reaction temperature restriction on the use of Cas12b in mammals [28].

Lastly, there is a variance in the number of constituents. Although the Cas9 system has been artificially reduced to two components, the Cas9 system in its natural state consists of three components, whereas both Cas12a and Cas12b are naturally composed of two components. Fewer components lead to greater adaptability and editing efficiency, as well as a broader application scope. Various action mechanisms result from the presence of distinct components. Cas9 protein is involved in dsDNA cleavage by HNH and Ruv-like domains, and it generates blunt DNA double-strand breaks close to the PAM site. Whether activated Cas12 cleaves ssDNA or dsDNA is determined by the RuvC domain. Depending on the RuvC domain’s dependence on Mg^2+^, the trans-cleaving activity of the Cas12 system can be modified by varying the reaction buffer [57]. Moreover, Ma et al. [58] discovered that the reaction buffer containing Mn^2+^ was more suitable for the LbCas12a system, whereas the fluorescence signal generated by the reaction buffer containing Mg^2+^ was 3.4–13.6 times greater. In addition to altering the divalent cations in the reaction system, additives like polyethylene glycol (PEG), glycerin, Triton X-100, bovine serum albumin (BSA), i-proline, etc., can increase the sensitivity of Cas12a in nucleic acid detection systems [59,60]. Cas12a nuclease generated sticky ends at the site of cleavage, whereas Cas12b nuclease cleavage resulted in a staggered break of 7 nucleotides at the cleavage site. A conserved aromatic residue site W355A in the mutant Cas12a REC2 domain increased the lytic activity of the Cas12a protein to target DNA [61]

In addition to single-molecule experiments, integrated DNA cleavage, and nanopore sequencing, the DNA cleavage activity and molecular dynamics of Cas12a have been investigated. The results showed that crRNA with a 20-nucleotide interspaced length was more likely to promote the cleavage of target sequences than crRNA with a 24-nucleotide interspaced length. In addition, TA-rich 7 nt DNA with the crRNA3’ terminal extension can significantly increase the sensitivity of detection, with a fluorescence signal that is 3.5 times greater than that of wild-type crRNA. AsCas12a does not benefit from crRNA. This sensitivity decreases as phosphorylation increases following extension [62].

Cas12a has a lower off-target rate than Cas9, which has generated widespread concern [63]. Specific interactions between target nucleotides and protein domains have been demonstrated, and protein sequence and structure optimization can enhance the targeting specificity of the CRISPR system during molecular detection. Wang and colleagues decreased the off-target rate by introducing point mutations at Cas9 residue K855A via sequence modification [49]. During the cleavage of the complementary sequence by Cas12a, the REC2 domain and the Nuc domain are close to one another, thereby narrowing the groove between the target strand and the RuvC active site [64,65,66]. These equivalent, additional target nucleic acid-binding domains found in the V-type CRISPR system are all capable of transmitting target chains to the active site of RuvC [67,68]. It appears that genetic modification of the REC2 domain can further reduce the off-target rate and increase the trans-cleavage activity of Cas12a based on the above results [64].

Cas14a, like Cas12, has the ability to bind to target nucleic acids and activate its ssDNA trans-cleavage activity, allowing it to be used for the molecular detection of target nucleic acids. Cas14a can only bind to ssDNA targets; consequently, the amplified enriched target nucleic acids must be treated with T7 exonuclease and one of the amplification primers must be modified with phosphothioacylation to ensure that T7 exonuclease only cuts one of the chains, leaving ssDNA target chains for Cas14A-mediated molecular detection [29].

### 3.4. Target RNA Binding and Cleavage

SaCas9, SpCas9, FnCas9, Cas13a, and Cas13b are all RNA-guided gene editing techniques present in the adaptive immune systems of numerous bacteria. Using specially engineered exogenous PAM-presenting oligonucleotides (PAMmers), SpCas9 can bind and cut specific RNAs while avoiding DNA sequences. This property enables the isolation of endogenous mRNA from the cytoplasm [69]. The disadvantages of SpCas9 nucleases are the large size of the protein and the need to link complex modifications. SpCas9 is over 1000 bases shorter than SaCas9, and its editing efficiency is comparable to that of SaCas9; however, the activity of SaCas9 is low. In addition, their utility in various molecular diagnostics is hindered by their individual flaws.

Cas13a and Cas13b are both composed of crRNA and Cas proteins, and the non-specific destruction of side-strand RNA by Cas13 systems results in the production of off-target. When Cas13 protein binds to target ssRNA, the central seed region of crRNA forms RNA double-stranded RNA, inducing conformational changes of Cas13 protein, activating catalytic sites of HEPN, and generating ribonuclease activity capable of non-specific cleavage of adjacent ssRNA [70]. The Cas13b system has generated two proteins, Csx27 and Csx28, that are related. Csx27 inhibits Cas13b-mediated RNA interference, while Csx28 enhances the cleavage activity of Cas13b [71,72]. In 2018, Silvana [73] identified Cas13d, a new class of Cas13 systems. Cas13d has several substantial advantages over other Cas13 subtypes: Cas13d possesses dual nuclease activity, allowing it to not only cut ssDNA but also mature crRNA. Consequently, PFS is not required to exert cleavage activity. These characteristics of Cas13d make it an excellent tool for spatiotemporal transcriptome engineering, nucleic acid detection, selective cleaving, tracing, and epigenetic regulation of RNA [74].

## 4. CRISPR/Cas Systems in Molecular Detection: Strategies and Applications beyond Genome Editing

### 4.1. Cas9 Protein

**Figure 2:**
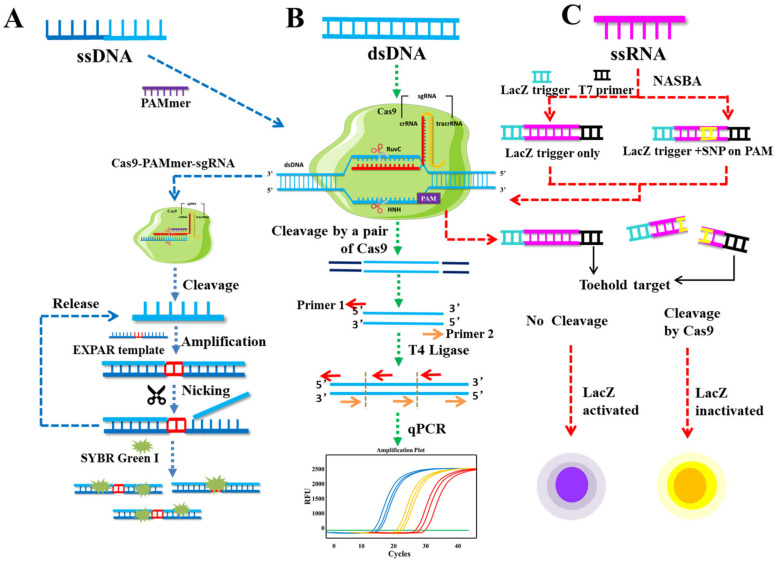
Schematic representation of CRISPR/Cas9 system. The Cas9 system can be used in combination with other amplification techniques to achieve the target cleavage activity. (**A**) CAS-EXPAR system. In the presence of PAMmer complementary to ssDNA, short fragments are obtained by specific cleavage activity of Cas9 protein and hybridized with EXPAR template to form dsDNA. The amplification products were nicked by Nease endonuclease and replaced with a new short fragment; the amplification process performs cyclically. Finally, the signal reporting of generating dsDNA was detected using SYBR Green I fluorescent dye. (**B**) CARP system. The target dsDNA is firstly cleaved by a pair of Cas9 complex proteins carrying specific sgRNAs; the short fragments are ligated by T4 ligase to form concatenated linear DNA, which is used as templates for subsequent amplification by a pair of reverse primers. (**C**) NASBACC system. ssRNA targets are reversely transcribed by the NASBA amplification system, and the resulting amplified products contain lacZ coding sequences. Cas9 then acts as an auxiliary sensor to identify base variations in the target sequence. Target sequences with PAM can be cut by Cas9, resulting in the failure of the switching target, while other sequences will remain intact and initiate the *LacZ* colorimetric effect.

In 2016, Cas9 was routinely used in eukaryotic cells to delete and modify DNA sequences because it is an effective, scalable, and fascinating genetic engineering tool [75,76]. By recognizing the PAM site on the target DNA, the complex formed by the Cas9 protein specifically binds and cuts the target DNA under the guidance of sgRNA. Before nucleotide amplification, the cleavage properties of the CRISPR/Cas9 system were frequently employed in the development of molecular assay technologies (Figure 2). This strategy offers two significant advantages: the enrichment of rare or low levels of nucleotide components and the consumption of large amounts of interfering nucleotides [77]. Classical techniques such as Depletion of Abundant Sequence by Hybridization (DASH) [78], CRISPR-mediated Ultrasensitive detection of target DNA (CUT-PCR) [79], and Finding low Abundant Sequences by Hybridization (FLASH) [80], all enrich low-content target fragments without thermal cycling or denaturing steps.

CRISPR/Cas9 was first introduced as a programmable biomolecular component for Zika virus typing in 2016 [81]. Based on the PAM sites that are unique to the American Zika virus, a strategy was devised that leads to Cas9 protein for the selective cleavage of the trigger sequence into a short fragment, as well as the inactivation of the toehold switch. In contrast, the other Zika virus lacking PAM sites was able to obtain a complete sensor-activated sequence following nucleic acid sequence-based amplification (NASBA), allowing for colorimetric detection due to the absence of Cas9 cleavage. In a few hours, the resulting paper-based sensor can perform single-base differential virus typing.

The CRISPR- or Cas9/gRNAs-associated reverse PCR (CARP) is another Cas9-based technology for detecting and typing viruses [82]. It comprises Cas9 complex protein carrying a pair of target-specific sgRNAs cleaved the sample DNA, and the Cas9-cleaved fragments were ligated into intramolecular circular or intermolecular concatenated linear DNA by T4 DNA ligase; the ligated DNA was amplified using a pair of reverse primers in the subsequent amplification step. The technique uses a three-step procedure to type and identify high-temperature HPV virus and can detect the target gene at concentrations as low as 0.2 ng.

In addition to the direct recognition and cleavage of specific sequences, CRISPR/Cas9 can influence the amplification of nucleotides to distinguish target from non-target sequences. The team led by Huang combined the cleavage activity of CRISPR/Cas9 with exponential amplification reaction (EXPAR) to develop the single-strand nucleic acid biosensing technique CAS-EXPAR [83]. The strategy involves cutting ssDNA or RNA at specific sites guided by PAMmer to liberate short fragments that serve as primers to initiate the EXPAR process. This generates a substantial amount of dsDNA and ssDNA amplicons. Due to EXPAR’s powerful amplification capability, the analytical sensitivity of CAS-EXPAR can reach 0.82aM. Regarding specificity detection, CAS-EXPAR can identify a single base mismatch at the cut site because the mismatch inhibits EXPAR’s primer extension, thereby terminating amplification. This technique has been applied successfully to the detection of *Listeria monocytogenes* mRNA and the determination of DNA-specific site methylation.

**Figure 3:**
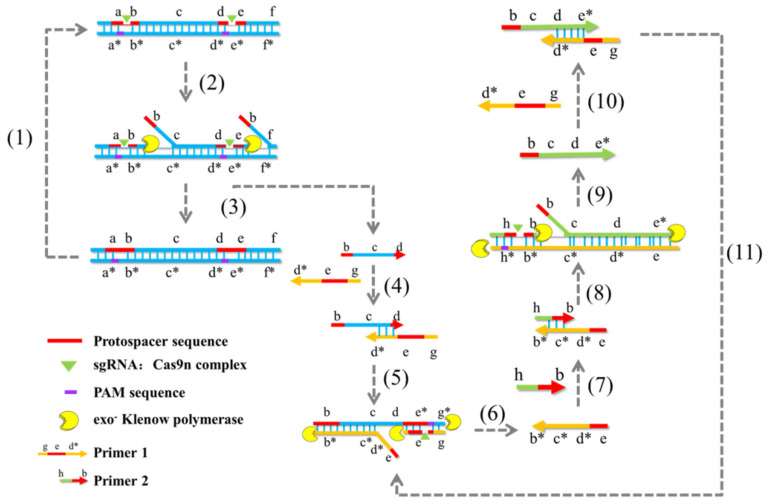
Schematic representation of Cas9nAR system for amplification of a DNA fragment.

By inactivating one of the HNH and RuvC nuclease domains of the Cas9 protein, single-strand cleavage of target dsDNA can be achieved. Cas9 nickase-based amplification reaction (Cas9nAR) (Figure 3) was developed based on this property [84]. The amplification reaction consists of two cycles. The first cycle removes the target ssDNA from the genome using sgRNA pairs, Cas9 nuclease mutants, and Klenow polymerase, while the second cycle amplifies the released ssDNA. At 37 °C, it is possible to detect single-base specific target sequences. Correspondingly, the accuracy may surpass 10 copies/μL. Although this technique has the advantage of not being limited by the length of target amplification, it shares the same disadvantages as the NASBA-CRISPR Cleavage technique (NASBACC), including a lengthy detection time, numerous reaction steps, and complex operation. Cas9 lost its ability to cut double-stranded DNA (dsDNA) when amino acid mutations rendered its HNH and RuvC nuclease domains inactive. This led to the production of the mutant Cas9 dCas9 protein. dCas9 retains the ability to target dsDNA selectively. By fusing other functional proteins to dCas9, a CRISPR/dCas9 detection system can be created. Multiple sgRNAs can direct dCas9 to bind simultaneously to several distinct target sites for parallel analysis [85]. For instance, the combination of a transcription suppressor or transcription activator with the dCas9 protein can inhibit or activate target site genes. To achieve the experimental purpose of Chromatin immunoprecipitation (ChIP) assay, nuclear localization signals and epitopes were introduced into the dCas9 system to label the target genes and their interacting proteins, thereby realizing the ChIP assay [86]. The peptide immobilization by Cas9-mediated self-organization (PICASSO) platform is a peptide sequence library that can fuse recombinant peptides with dCas9 protein and utilize sgRNA sequences as barcodes [87]. Briefly, PICASSO could affix a custom-made collection of proteins to a modified, non-catalytic Cas9 protein (dCas9), after which the decatalyzed Cas9 would bind to the DNA sequence without cleaving it, followed by labeling using the unique sgRNA sequence. The dCas9 peptide complex will eventually self-assemble with the complementary DNA sequence of the sgRNA. Thus, with PICASSO, the proteins in the complex self-assemble with the corresponding DNA sequence whenever the DNA molecule is positioned at a particular location on the surface of the microarray. The resulting DNA template protein microarray can rapidly identify antibodies in clinical samples, enabling the simultaneous identification of thousands of antibodies. This technology overcomes many of the disadvantages of other protein detection platforms, such as high cost, time, and effort, and can be used for the rapid customization of DNA-scaffolded protein libraries or biological materials. For miRNA classification detection, Rolling Circle Amplification (RCA) and split-horseradish peroxidase (HRP) techniques were combined with the CRISPR/dCas9 detection system. In this assay, the target miRNA was combined with a specific probe in the shape of a dumbbell, and the double-stranded stem region of the probe was opened to form a ring probe, which was amplified by rolling ring under the action of phi29 DNA polymerase. Numerous repeated and uninterrupted single-stranded DNA sequences were produced. Due to the complementary pairing region within the sequence, a large number of regular stem-ring structures are capable of forming through complementary pairing. Subsequently, dCas protein fused with split-HRP report fragments was added, and under the guidance of sgRNA, the stem-ring structure of the amplified product was identified, after which the separated HRP report fragments were aggregated to form active HRP protein. The HRP protein then demonstrated catalytic activity to transform yellow substrate TMB (Tetramethylbenzidine) into a blue product. Accordingly, a low-cost and effective miRNA detection platform was successfully developed [88]. The detection platform has a single-base specificity, can detect target miRNA with a sample concentration as low as 35.4 amol, and can produce visual determination results based on the color change of the reaction solution. Additionally, there are constraints on the application of this strategy. For example, the protein concentration, optimal RCA time, and dCas9 binding time should be determined further for various miRNAs, and the strong dependence of dCas9 on the PAM site determines that two suitable target sequences are close enough to be effectively detected by the HRP reporter. Currently, the dCas9 system is primarily employed to validate phenotypic analyses of epigenetics, cell differentiation, and disease progression [89,90].

### 4.2. Cas12 Protein

Cas12 possesses trans-cleavage activity and is utilized as one of the major branches of CRISPR systems following nucleic acid amplification. As a result of their recognition of specific sequences, Cas protein complexes generate an abundance of reporter cleavages that improve the specificity and sensitivity of molecular detection. The sgRNA “seed sequence” of Cas9 nuclease consists of the first 7–8 bases, which are susceptible to mistargeting due to the mismatch of later bases, whereas the “seed sequence” of Cas12 nuclease, specifically Cas12a nuclease, consists of the first 18 bases of sgRNA. CRISPR/Cas12a is more accurate because it checks each base pair more thoroughly [91]; it is widely applicable for pathogen detection and genomic SNP identification.

**Figure 4:**
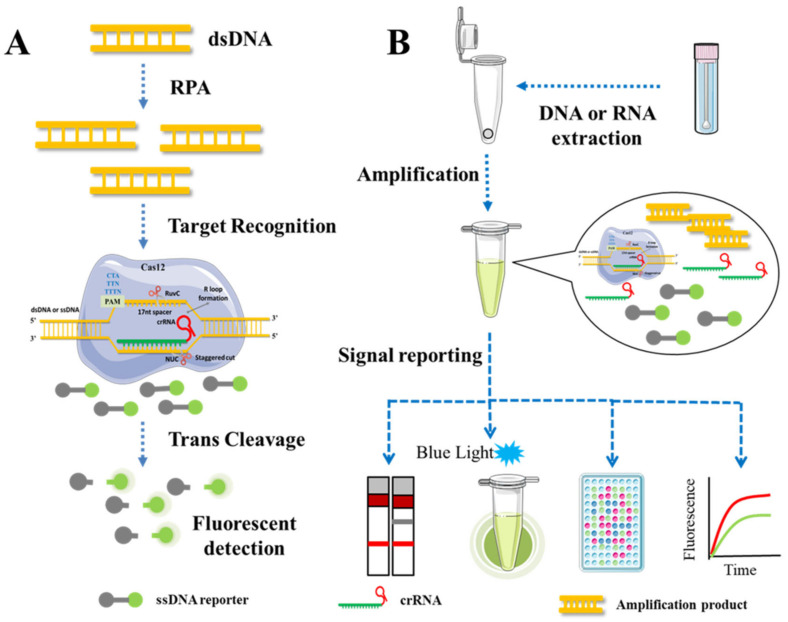
Schematic representation of CRISPR/Cas12 system-based molecular method. (**A**) DETECTR system. (**B**) The qualitative and quantitative analysis, based on CRISPR/Cas12 system. Following DNA or RNA extraction, the target sequences are amplified by the PCR or RT-PCR, the trans-cleavage activity of Cas12 protein is activated by the target ssDNA or dsDNA, and the fluorescence signal can be read according to the detection requirements.

In the development of a CRISPR/Cas12 detection platform, isothermal amplification techniques such as LAMP, Recombinase Polymerase Amplification (RPA), and RCA are frequently used to amplify target sequences due to the similar reaction temperature range of the Cas12 protein. The combined detection technology possesses both a high degree of specificity and efficiency. As an illustration, DNA Endonuclease-Targeted CRISPR Trans Reporter (DETECTR) [92] (Figure 4) and Low-cost Multipurpose highly Efficient System (HOLMES) [55], replaced Cas12a with Cas12b and upgraded to HOLMES v2 [54]. Jiao et al. developed the first Cas12a-based visual detection platform capable of detecting five distinct apple RNA viruses or viroids simultaneously by combining CRISPR/Cas12a with reverse transcription amplification [31]. The entire detection process is conducted at body temperature (36–37 °C) and takes no more than one hour from sample collection to result interpretation. This straightforward method is applicable to samples that contain natural inhibitors and are incompatible with RT-PCR, and it can be used quickly and accurately to detect viruses in the field, aiding in the early suppression of viral transmission.

In conjunction with CRISPR/Cas and isothermal amplification, the detection process frequently encounters lid-opening-induced aerosol pollution. Typically, physical separation resolves the aforementioned problems. The isothermal amplification system and CRISPR/Cas system are separated within a single tube, or multiple isolation zones are created by modifying the tube cap. Based on the DETECTR technology, Wang and his colleagues optimized the Cas12aVDet detection platform, which has the same sensitivity as DETECTR [93]. The RPA reaction system was placed at the bottom of the tube, and freeze-dried CRISPR/Cas12a powder was placed on the tube cap. Upon completion of the amplification reaction, the product was centrifuged and uniformly combined with the CRISPR/Cas12a system on the tube cap. The detection procedure required only a transportable heater and a blue-light display. Subsequently, the addition of a layer of mineral oil to the reaction liquid at the bottom of the tube can prevent the reaction solvent from evaporating and better insulate the two systems [94]. Although physical isolation can result in a single-tube reaction, multi-step operations make it challenging to detect the reaction in real time and are time-consuming. To integrate the isothermal amplification reaction and CRISPR/Cas system into a single tube for homogeneous reaction, it is necessary to determine the compatibility between the two systems, including reaction temperature, buffer composition, pH, and metal ions.

When CRISPR/Cas12a was combined with RT-RPA to develop a single-tube homogeneous thermostatic reaction, the sensitivity of RNA with a low copy number (<1000) was poor because CRISPR/Cas12a bound to both the amplification products and target chains of RT-RPA, inhibiting the RPA reaction. Ding [95] designed two groups that recognize CRISPR/Cas12a without PAM and only identify ssDNA produced during the amplification of RPA, thereby reducing the impact on RPA. Feng [96] accelerated the dissociation of RNA from cDNA in the reaction system by using reverse transcriptase with RNase H function or by adding RNase H directly, thereby liberating cDNA. For the two systems, Lu [97] designed new crRNA and reverse transcription primers for SPAMC compatibility (suboptimal PAM). In this method, the suboptimal PAM site was utilized to recognize crRNA and reduce CRISPR/ability Cas12a’s to bind to dsDNA, in order to preserve the amplification efficiency of RPA. RPA amplification products were detectable in 2 min, and the detection threshold was comparable to RT-PCR. Researchers proposed a-crRNA-enhanced CRISPR system (meCRISPR) without expansion for the detection of adulteration in meat products [32]. The total concentration of crRNA was 200 mM, and it was distributed uniformly among all crRNAs. When the ratio of Cas12a to crRNA was maintained at 1:2, the best detection results were observed. Compared to a single crRNA, the combination of four crRNAs significantly increased sensitivity. The detection limit for pork DNA extraction was as low as 1.13 ng/μL and 5% (*w*/*w*) for pork/beef mixed species.

The CRISPR/Cas12 detection technology can not only rely on the DNA nuclease activity mediated by crRNA but it can also be combined with other research fields to create a new interdisciplinary platform. In the published report, scientists constructed a dumbbell-shaped dopamine–magnetic bead compound probe by modifying the magnetic bead and the dopamine-linked liposome at both ends of ssDNA. Cas12a degrades the double dumbbell probe by cleaving ssDNA when the sgRNA binds to the target HPV16, and the photoelectrochemical biosensor detects the photocurrent by utilizing the trans-cleavage activity of Cas12a [98]. In addition to the typing detection of various viruses, the CRISPR/Cas12a system has been used to develop biosensors for exosomes [99], small molecular compounds [100,101], melamine [102], and other fields. The development strategy for this type of biosensor is as follows: When a target-mediated binding event (such as nucleic acid hybridization or protein interaction) occurs, biotin modification or conformational modification of the template sequence induces sgRNA to form a complex with Cas12a protein to recognize the target sequence and activates Cas12a’s target specific cleavage and trans-cleavage activity for ssDNA. A fluorescence signal proportional to the target sequence dose is then generated. This type of biosensor has a short detection time, high sensitivity and specificity, and the ability to detect substances rapidly at room temperature.

### 4.3. Cas13 Protein

**Figure 5:**
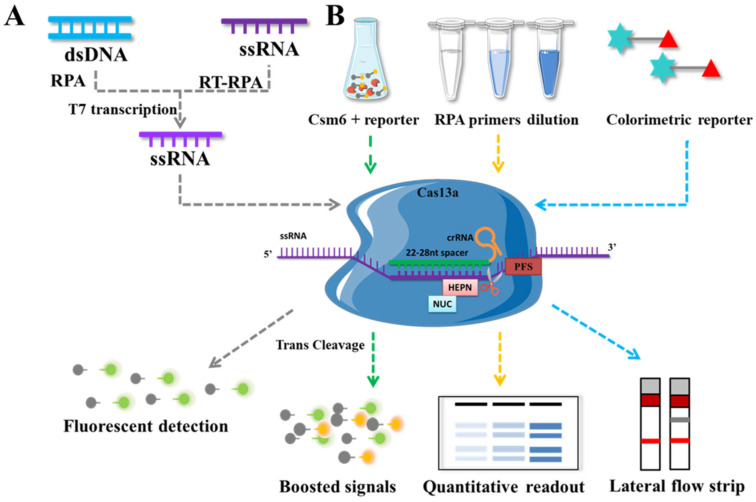
Schematic representation of CRISPR/Cas13 system-based molecular method. (**A**) SHERLOCK system. (**B**) The quantitative detection combined with CRISPR/Cas13 system, Csm6 is a type III auxiliary CRISPR-effector nuclease that can conjugate its reporter signal to Cas13a for boosted signals; quantitative detection can be achieved by gradient dilution of RPA primers; the colorimetric reporter combining lateral flow strip for visualization result.

Cas13 is an RNA-guided RNA endonuclease that can specifically cleave ssRNA under the guidance of crRNA and retain non-specific endonuclease activity to cleave additional non-target ssRNAs after the cleaving is complete. East Seletsky employed LbuCas13a for the first time in 2016 to detect phage λRNA and endogenous β-actin mRNA. The target ssRNA activates the trans-cleavage activity of Cas13a, which degrades the fluorescence-quenched-group-labeled ssRNA, thereby releasing a fluorescence signal and enabling rapid detection with detection limits as low as 1–10 pM [11]. The majority of existing CRISPR/Cas13-based detection technologies are based on SHERLOCK [103] (Figure 5): after the target sequences are pre-amplified by recombinant polymerase RPA or RT-RPA, they are transcribed by T7 RNA polymerase to form ssRNA, which causes Cas13 to cleave the fluorescent probe of ssRNA and release fluorescent groups. The entire reaction procedure allowed for the rapid detection of RNA and DNA at 37 °C and amol concentrations. SHERLOCK V2 is improved as a result of this research [104], adding four advantages: (1) The realization of a single tube quadruple detection; (2) By adjusting the RPA primer to control the process of exponential amplification, quantitative detection at the concentration level of amol was achieved; (3) Cascade activation of Csm6 nuclease activity triggered by side chain cleavage of Cas13a enhanced the sensitivity of the assay system; (4) Target sequences as low as 2 aM can be visualized within 90 min.

The signal amplification capacity of CRISPR-based trans-cleavage activity is determined by enzyme digestion kinetics parameters, which are indispensable for determining the detection limits of various Cas nucleases. Although CRISPR/Cas13a can directly recognize ssRNA and still cut probes to generate signals at room temperature, the enzyme digestion kinetics parameters of Cas13a make it difficult to detect low-copy RNA. Fozouni [105] designed multiple CRISPR/Cas13a to simultaneously recognize different sites of SARS-CoV-2 RNA and activate the cutting activity of multiple groups of CRISPR, thereby significantly improving the signal amplification efficiency. A highly sensitive fluorescence detection instrument was used concurrently to detect weak fluorescence signals. A CRISPR-based amplification-free digital RNA detection method (SATORI) for microdrops was proposed by Shinada [106] as a second CRISPR/Cas13a-based RNA detection strategy. Cas13a, target RNA, and fluorescently labeled substrate chains were dispersed into 120,000 chambers containing 3 fL. To facilitate the activation of CRISPR/Cas13a, the local concentration of RNA in uniformly dispersed droplets was increased, and fluorescent groups produced by enzyme digestion were gathered in the microdroplets to concentrate the fluorescence signal. Correspondingly, the ability of CRISPR/Cas13a to detect target RNAs can be improved by enhancing the signal generation procedure and detection equipment.

Cas13 is a nucleic acid detection platform with aM sensitivity and single base specificity that is appropriate for the development of multifunctional, rapid, convenient, and cost-effective nucleic acid detection technology. This is essential for point-of-care testing, epidemiological surveillance, and pathogen detection in settings with inadequate infrastructure.

### 4.4. Cas14 (Cas12f) Protein

Cas14 systems are the last effective CRISPR systems ever discovered. It is comprised of 400 to 700 amino acids, is half the size of the regular Cas9 protein, and can be combined with fluorescent markers that bind to single-stranded DNA fragments. When Cas14 binds to its target DNA sequence and initiates DNA cleavage, it also cleaves single-stranded DNA fragments attached to the fluorescent marker to produce a fluorescent signal [30]. Cas14a displayed a strong preference for longer fluorescent ssDNA substrates during the incubation process with the target ssDNA [107]. While CRIPSR/Cas14 can recognize target sequences without a PAM sequence, it is extremely sensitive to mismatches in the target region. The Cas14 system is, thus, ideally suited for detecting single nucleotide polymorphisms. DETECTR–Cas14 technology, developed with the combination of RPA technology and Cas14, has greater specificity than the previous DETECTR–Cas12 technology, and has been applied successfully to the typing of SNP genes [29]. As the substrate for the Cas14 reaction, DNA is more stable and has fewer environmental requirements. It facilitates the advancement of nucleic acid targeted editing, virus detection, and nucleic acid detection through protein purification.

## 5. Conclusions

With its editable, highly specific nucleic acid recognition capability and cis- and trans-cutting capabilities, the CRISPR/Cas system has been developed into an adaptable and universal molecular detection tool. Target amplification, binding (with or without cutting), fluorescence signal analysis, etc., are the fundamental processes underlying current CRISPR/Cas-based detection techniques (Table 3). As an exogenous gene-editing system, however, additional scientific validation is required for the potential off-target effects of the CRISPR/Cas system, the toxic effects of CRISPR components, and the sequence-based limitations of sgRNA design. Although continuous optimization of sgRNA length, construction of Cas protein variants, and the development of novel single-base and double-base editors can improve the aforementioned issues and increase the efficiency of gene editing and nucleic acid detection to a certain extent, CRISPR/Cas-based molecular detection technology still faces numerous challenges and opportunities. The following are examples: (1) Developing the CRISPR/Cas system in conjunction with other nucleic acid amplification methods to improve the detection of extremely low concentrations of nucleic acid; (2) concentrating on the rapid and convenient detection technology based on single tube homogeneous isothermal amplification, so that nucleic acid detection results can be visualized, such as test strips, portable ultraviolet lamp, color change, etc.; (3) developing a single tube constant temperature non-fluorescent detection method using isothermal amplification and CRISPR, such as electrochemical [108] or colorimetric detection method [109], to improve the matrix tolerance of non-fluorescent detection system; (4) developing new storage and use methods, including paper-based lyophilized powder reagents [81], and avoiding reducing the activity of reaction reagents during storage, transportation, and use; (5) developing more convenient and efficient sample pretreatment methods, reducing the operation process and time, while increasing the suitability for different types of samples; (6) developing high-throughput detection methods, and multi-fluorescent path detection probes, for use in conjunction with high-throughput detection platforms. Additionally, by conducting in-depth research on the cutting function of the Cas nuclease, more exponential nucleic acid amplification systems based on the same Cas nuclease will be incorporated into the development and establishment of single-tube homogeneous isothermal detection methods.

## Figures and Tables

**Table 1:** Subtypes and proteins contained in CRISPR/Cas systems.

Class	Types	Subtypes	Effector Module	Spacer Integration
Class 1	I	I-A,I-B,I-C,I-G,I-D,I-E,I-F1,I-F3,I-F2,	Cas3″,Cas5,Cas6,Cas7, Cas8, Cas11, Cas10	Cas1,Cas2,Cas4
	III	III-A,III-D,III-E,III-F,III-C,III-B	Cas5,Cas7,Cas10,Cas11, Csx19	Cas1,Cas2
	IV	IV-A,IV-B,IV-C	Cas5,Cas7,Cas8, Cas11	Cas1,Cas2
Class 2	II	II-A,II-B,II-C1,II-C2	Cas9	Cas1,Cas2,Cas4
	V	V-A,V-E,V-B1,V-B2,V-I,V-H,V-C, V-D,V-F1,V-F1(V-U3),V-F2,V-U2, V-U4,V-F3,V-U1,V-G,V-K(V-U5)	Cas12	Cas1,Cas2,Cas4
	VI	VI-A,VI-D,VI-C,VI-B1,VI-B2	Cas13	Cas1,Cas2

**Table 2:** sgRNA characterizations associated with target activity.

Categories	Efficient Characterizations	Inefficient Characterizations
Nucleotide content	A count	U, G count
	A in the middle of sgRNA	GG, GGG count
	AG, CA, AC, UA count	UU, GC count
Position-specific nucleotides	G in position 20	C in position 20
	A in position 20	U in position 17–20
	Purines in position 19	G in position 16
	C in position 18	T in PAM (TGG)
	C in position 16	G in position + 1 (NGGG)
	C in PAM	
Structural characterizations	GC in first ten bases	GC content > 80% or GC content < 35%
	GC in position 4–8	Stable self-folding
	GC in position 15–20	Stable DNA/RNA duplex
	* Low T_m_ in the middle of sgRNA	
	Accessibility in positions 18–20	
	GG at 5′ end of sgRNA	
	Extension at 5′ end of tracrRNA	
Genomic context	Target near N-terminus of coding seq	Target in 5′ or 3′ UTR
	High Cas9-sgRNA concentration	Low Cas9-sgRNA concentration
	Target chromatin more open	Target in introns
Motifs	NGG PAM	Poly-N,TT-motif, GCC-motif

* T_m_: melting temperature, UTRs: untranslated regions.

**Table 3:** Major characteristics of the CRISPR/Cas-based detection platforms.

Experimental Method	Effector	Amplification	Sensitivity	Specificity	Quantitative Detection	Multiple Detection	Read Out	Time	Sample	Reference
RCA	Sp-dCas9	RCA	fM	1 nt	Yes	No	Colorimetric	<4 h	RNA	[88]
NASBACC	SpCas9	NASBA	fM	1 nt	No	No	Colorimetric	≈3 h	RNA	[81]
PC reporter	Sp-dCas9	PCR	1 copy	/	No	No	Bioluminescent	10 min after PCR	DNA	[86]
CAS-EXPAR	SpCas9	EXPAR	aM	1 nt	No	No	SYBR Green I	<1 h	DNA/RNA	[83]
Cas12aVDet	Cas12a	RPA	10 aM	1 nt	No	No	Fluorescent signal (FAM)	30 min	DNA	[93]
RPA-Cas12a-FS	Cas12a	RPA	10 copies	1 nt	No	No	Fluorescent signal (HEX)	45 min	DNA	[33]
HOLMES	LbCas12a	PCR, RT-PCR	aM	1 nt	No	No	Fluorescent signal (HEX)	≈1 h	DNA/RNA	[55]
DETECTR	LbCas12a	RPA	aM	6 nt	No	No	Fluorescent signal (FAM)	≈2 h	DNA	[92]
MeCas12a	LbCas12a	RT-RAA, qPCR	5 copies	1 nt	No	No	Fluorescent signal	30 min	DNA/RNA	[58]
HOLMES v2	AacCas12b	LAMP, RT-LAMP, Asymmetric PCR	aM	1 nt	Yes	No	Fluorescent signal (HEX, FAM)	≈1 h	DNA/RNA	[54]
STOPCovid.V2	AapCas12b	LAMP	100 copies				Fluorescent	≈1 h	RNA	[110]
SHERLOCK	LwCas13a	RPA	aM	1 nt	No	No	Fluorescent signal (FAM)	2–5 h	DNA/RNA	[103]
SHERLOCK v2	LwCas13a CcaCas13b PsmCas13b	RPA	zM	1 nt	Yes	Yes	Fluorescent signal (FAM, TEX, Cy5, HEX); Colorimetric (Gold-NP, anti-FAM, Abs)	0.5–43 h	DNA/RNA	[104]
HUDSON + SHERLOCK	LwCas13a	RPA	aM	1 nt	No	No	Fluorescent signal (FAM); Colorimetric (Gold-NP, anti-FAM, Abs)	<2 h	DNA/RNA	[111]
Cas14SDA	Cas14a	SDA	680 fM	1 nt	Yes	No	Fluorescent signal (FAM)	<1 h	RNA	[36]
DETECTR-Cas14	Cas14a	RPA	aM	2 nt	No	No	Λex: 485 nm; Λex: 535 nm	≈2 h	DNA	[29]

## Data Availability

No new data were created or analyzed in this study. Data sharing is not applicable to this article.
